# The changes of serum inflammatory cytokines in patients with hemiplegia after ischemic stroke and the rehabilitation effects

**DOI:** 10.5937/jomb0-55968

**Published:** 2025-08-21

**Authors:** Hongxia Li, Yunqi Lai

**Affiliations:** 1 Zhejiang Rehabilitation Hospital, Three Rehabilitation Areas/Rehabilitation Zone 3, Hangzhou, Zhejiang Province, China

**Keywords:** ischemic stroke, hemiplegia, rehabilitation, neuroplasticity, inflammatory cytokines, precision exercise therapy, motor recovery, ishemijski moždani udar, hemiplegija, rehabilitacija, neuroplastičnost, inflamatorni citokini, terapija preciznim vežbama, motoricki oporavak

## Abstract

**Background:**

Acute ischemic stroke (AIS) often leads to hemiplegia, significantly impairing neurological function, motor ability, and daily life activities. Early precision exercise rehabilitation has emerged as a promising intervention to enhance recovery. This study evaluated its effectiveness in improving neurological function, gait performance, and self-care ability and reducing inflammatory response in hemiplegic patients.

**Methods:**

This retrospective cohort study included 230 patients with hemiplegia due to AIS, admitted within 72 hours of onset. Patients were divided into an Early Rehabilitation Group (EG, n = 132) and a Conventional Rehabilitation Group (CG, n = 98) based on the intervention received. The EG underwent early precision exercise rehabilitation, integrating neurofunctional training, motor-evoked potential (MEP) therapy, and functional electrical stimulation (FES), while the CG received traditional rehabilitation. The effectiveness was assessed using the National Institutes of Health Stroke Scale (NIHSS), Wisconsin Gait Scale (WGS), and Activities of Daily Living (ADL) scores. Serum inflammatory markers (TNF-a, hs-CRP IL-6, IL-18) were also measured before and after treatment.

**Results:**

The EG demonstrated significantly more significant improvements in NIHSS (5.85± 1.31 vs 7.03± 2.54, P< 0.05), WGS (24.81± 3.06 vs. 31 .96 ± 4.62 , P< 0.05), and ADL scores (63.08± 4.93 vs. 51 .78 ± 6.34 , P< 0.05) compared to the CG. Walking frequency and speed were also higher in the EG (P< 0.05). Inflammatory markers significantly decreased post-treatment in the EG (TNF-a: P< 0.05, hs-CRP: P< 0.05, IL-6: P< 0.05, IL-18: P< 0.05), suggesting a reduction in systemic inflammation.

**Conclusions:**

Early precision exercise rehabilitation significantly enhances neurological function, motor ability, and self-care capacity, reducing inflammatory response in hemiplegic AIS patients. These findings support its integration into clinical stroke rehabilitation protocols.

## Abstract (sr)

**Uvod:**

Akutni ishemijski moždani udar (AIS) često dovodi do hemiplegije, značajno narušavajući neurološku funkciju, motoričke sposobnosti i aktivnosti iz svakodnevnog života. Rana precizna vežba rehabilitacija se pojavila kao intervencija koja obećava u smislu poboljšanja oporavka. Ova studija je procenila njenu efikasnost u poboljšanju neurološke funkcije, performansi hoda i sposobnosti samostalne brige, kao i u smanjenju inflamatornog odgovora kod pacijenata sa hemiplegijom.

**Metode:**

Ova retrospektivna kohortna studija obuhvatila je 230 pacijenata sa hemiplegijom uzrokovanom AIS-om, primljenih u roku od 72 sata od početka simptoma. Pacijenti su podeljeni u grupu rane rehabilitacije (EG, n = 132) i grupu konvencionalne rehabilitacije (CG, n = 98) na osnovu primljene intervencije. EG je prolazila kroz ranu rehabilitaciju preciznim vežbama, koja je uključivala neurofunkcionalni trening, terapiju motoričkog evociranog potencijala (MEP) i funkcionalnu elektricnu stimulaciju (FES), dok je CG dobijala tradicionalnu rehabilitaciju. Efikasnost je procenjena pomoću Nacionalne skale za moždani udar (NIHSS), Viskonsinske skale hoda (WGS) i Skora aktivnosti svakodnevnog života (ADL). Serumski inflamatorni markeri (TNF-a, hs-CRP IL-6, IL-18) mereni su pre i posle tretmana.

**Rezultati:**

EG je pokazala značajno veća poboljšanja u NIHSS (5,85± 1,31 naspram 7 ,0 3± 2,54, P< 0,05), WGS (24,81± 3,06 naspram 31,96 ± 4,62 , P< 0,05) i ADL skorovima (63,08± 4,93 naspram 51 ,78 ± 6,34 , P< 0,05) u poređenju sa CG. Frekvencija i brzina hoda takođe su bile veće u EG (P< 0,05). Inflamatorni markeri su značajno opali nakon tretmana u EG (TNF-a: P< 0,05, hs-CRP: P< 0,05, IL-6: P< 0,05, IL-18: P< 0,05), što ukazuje na smanjenje sistemske upale.

**Zaključak:**

Rana rehabilitacija preciznim vežbama značajno poboljšava neurološku funkciju, motoričke sposobnosti i kapacitet za samostalnu brigu, smanjujući inflamatorni odgovor kod pacijenata sa hemiplegijom izazvanom AIS-om. Ovi nalazi podržavaju njenu integraciju u kliničke protokole rehabilitate nakon moždanog udara.

## Introduction

Ischemic stroke is a common and highly disabling neurological disease among the middle-aged and elderly population. In China, with the intensification of population ageing, the incidence and mortality of ischemic stroke are increasing year by year [1] [2]. Ischemic stroke is usually caused by blockage of blood vessels in the brain, leading to brain tissue hypoxia and necrosis and damaging the brain's motor, sensory, language, and cognitive functions [3]. Clinically, the manifestations of ischemic stroke are diverse, with the most common being hemiplegia, that is, the loss of motor function on one side of the body, and patients often accompany varying degrees of limb paralysis, sensory disorder, and speech difficulties. As the physiological functions of patients with ischemic stroke gradually recover, the reshaping of the nervous system still faces visible challenges, and many patients still face long-term limb dysfunction even after acute treatment [4]. Therefore, how to effectively improve the sequelae of ischemic stroke, especially the limb function of patients with hemiplegia, has become a focus of clinical research and treatment.

Early rehabilitation training, as an important part of the treatment of ischemic stroke, has received widespread attention in recent years [5]. Early rehabilitation training aims to help patients recover more autonomous activity quickly after stroke by improving cerebral blood flow, promoting neural repair, and functional reconstruction [6] [7]. Rehabilitation training usually includes physical, occupational, speech, and exercise training, covering comprehensive intervention measures for motor, cognitive, and language functions. Studies have shown that early rehabilitation can effectively promote the recovery of patients' motor function, improve the quality of life (QoL), and reduce the incidence of complications. Especially in patients with hemiplegia, appropriate rehabilitation training can visibly reduce muscle atrophy and joint contracture caused by limb inactivity, improve the autonomous activity ability of limbs, and the ability to take care of daily life [8] [9]. In addition, early rehabilitation training can regulate neural plasticity and promote the reconstruction of neural networks, which is crucial for long-term functional disability improvement after stroke.

In addition to rehabilitation training, an increasing number of studies have focused on the role of serum inflammatory cytokines in the late rehabilitation process of ischemic stroke in recent years [10]. Studies have found that the acute-phase inflammatory response induced by ischemic stroke not only exacerbates brain damage but may also affect neural repair and functional recovery during the recovery period. Inflammatory cytokines such as interleukin-6 (IL-6), tumour necrosis factor-α (TNF-α), and high-sensitivity C-reactive protein (hs-CRP) increase in the body after stroke, and these inflammatory factors affect the survival, repair, and functional recovery of nerve cells by regulating the immune system's response to damage [11]. IL-6, in particular, contributes to the body's immune response and plays a crucial role in cerebrovascular permeability, blood-brain barrier disruption, and neuronal apoptosis [12] [13]. Consequently, changes in inflammatory cytokine levels are regarded as significant biomarkers for evaluating the rehabilitation outcomes and prognosis of patients with ischemic stroke. Although early rehabilitation training has achieved visible effects in promoting the functional recovery of patients with hemiplegia, how to combine other adjuvant treatment methods to improve the rehabilitation effect further is still an important direction of current clinical research. In recent years, more and more studies have begun to explore comprehensive treatment strategies that combine early rehabilitation training with other treatment methods, especially combining immune regulation and inflammation control with rehabilitation training, which has become a new research trend [14] [15]. Studies suggest controlling inflammatory responses can accelerate neural repair and improve functional recovery. With the development of technology, new adjuvant treatment methods such as drug treatment, neural stimulation therapy, and exercise biofeedback have gradually been introduced into the field of stroke rehabilitation, providing a more comprehensive rehabilitation plan for patients with hemiplegia [16]. Based on this background, exploring the rehabilitation effect of early rehabilitation training combined with adjuvant treatment on the limb-nervous function of patients with hemiplegia caused by ischemic stroke, especially the impact on changes in serum inflammatory cytokine levels, is of great clinical significance.

This article aims to evaluate the effect of adjuvant treatment with early rehabilitation training on the limb-nervous function recovery of patients with ischemic stroke and hemiplegia and to explore the correlation of changes in serum inflammatory cytokine levels. By systematically evaluating the changes in patients' limb function, neural function recovery, and inflammatory cytokine levels, it hopes to provide a more scientific basis for the rehabilitation treatment of patients with ischemic stroke and hemiplegia, help to formulate more reasonable rehabilitation treatment plans and improve the rehabilitation effect of patients. This article also compared the efficacy under different rehabilitation training modes, providing a reference for early intervention and personalised treatment in clinical practice.

## Materials and methods

### Subjects

A retrospective inclusion of 230 hemiplegic patients diagnosed with AIS at Zhejiang Rehabilitation Hospital from January 2021 to October 2024 was conducted. There were 125 males and 105 females, with an age range of 48-73 years. The Zhejiang Rehabilitation Hospital Ethics Association approved this experiment's implementation.

Inclusion criteria: (1) The onset time of AIS symptoms in patients did not exceed 24 hours upon admission; (2) Admission within 72 hours of acute onset; (3) Clear neurological localisation signs; (4) Neurological function deterioration within 2-3 days after onset.

Exclusion criteria: (1) Patients with severe liver, kidney, heart, or lung diseases, etc.; (2) Those who had received other medications or treatment methods in addition to standard treatment; (3) Patients involved in other stroke-related interventional clinical trials or studies; (4) Deterioration of condition with new infarction or bleeding; (5) History of mental illness.

### Case grouping and treatment methods

Based on different treatment plans, the cases were grouped: EG (132) and CG (98).

The CG's treatment plan primarily focused on traditional rehabilitation training, which mainly included physical, occupational, and speech therapy. Physical therapy mainly consists of passive and active motion training and joint mobility training to improve patients' limb activity ability, restore joint motion function, and prevent muscle atrophy and joint contracture due to long-term bed rest or insufficient exercise. Occupational therapy focuses on restoring the ability to take care of daily life, such as training in basic movements like eating, dressing, and grooming, helping patients gradually regain the ability to live independently. The CG underwent standard speech therapy for patients with language function impairment to help improve pronunciation, language comprehension, and expression abilities. The frequency of rehabilitation training was once a day, lasting 30 to 60 minutes per session, focusing on restoring basic functions and lacking personalised interventions tailored to individual patient conditions.

Compared to the CG, the EG's treatment plan added precision exercise rehabilitation to traditional rehabilitation training, emphasising individualisation, quantification, and early intervention. The EG began precision exercise rehabilitation within the acute phase, usually 72 hours after admission. Personalised exercise plans were formulated based on the patient's specific conditions, such as the degree and location of motor function impairment, focusing on lower limb weight-bearing training, upper limb range of motion training, and functional movement training to promote muscle strength recovery in the affected limbs and prevent muscle atrophy and joint stiffness due to insufficient exercise. In addition, the EG also adopted modern neuroscience theory-based neurofunctional training, using methods such as motor evoked potential (MEP) technology and mirror therapy to promote cortical neural plasticity and reshape, accelerating neural repair processes. To improve treatment effects, the EG also used intelligent rehabilitation equipment, such as gait training robots, intelligent rehabilitation gloves, and virtual reality (VR) technology, which assist patients in more precise movement training through real-time feedback and data analysis, thereby improving rehabilitation efficiency.

Furthermore, the EG introduced functional electrical stimulation therapy (FES), which promotes muscle contraction through electrical stimulation to improve muscle strength and motor function in the affected limbs. The frequency of rehabilitation training in the EG was higher, usually twice daily, with each training session lasting 40 to 80 minutes. Additionally, the EG conducted comprehensive assessments twice a week, including motor ability, neurological function, and self-care in daily life, and the assessment results were used to adjust treatment plans to ensure individualisation and adaptability dynamically. The EG's treatment also adopted an interdisciplinary team cooperation model, with rehabilitation physicians, physical therapists, occupational therapists, and speech therapists working together to formulate and implement comprehensive patient rehabilitation plans, ensuring thorough and systematic treatment.

### Observational indicators

- (1) Clinical data such as the paralysed parts and onset time of patients were organised.
- (2) The NIHSS scores of all subjects were collected. NIHSS is a widely used neurological function assessment tool for people with AIS, aiming to help doctors evaluate the severity, prognosis, and treatment effects of stroke by quantifying the degree of neurological deficit. The scale includes 11 assessment items involving consciousness level, language ability, facial muscles, limb movement, visual function, eye movement, etc., with each item scored based on the symptoms, ranging from 0 to 4 points, with a maximum total score of 42 points. The higher total score means a more severe neurological deficit.
- (3) The WGS scores of all subjects were collected. This scale assesses gait function, mainly judging the stability, stride, speed, coordination, and fluidity of gait. The scoring content usually includes balance ability, walking speed, etc., with each item scored from 0 to 3 points, 0 points indicating normal and 3 points indicating severe disorder. The total WGS score reflects the severity of gait disorders. It is commonly used to assess gait issues in neurological diseases or the elderly population, providing a reference for treatment and rehabilitation.
- (4) The ADL scores of all subjects were collected. ADL is a scale adopted to assess an individual's daily ability to care for themselves, commonly adopted to determine independence and functional disability. ADL scores usually include several basic life activities, such as eating, dressing, grooming, mobility, and selfcare, with each activity scored based on whether the people can complete it independently or needs help from others. The score is usually from 0 to 4 points, with 0 points indicating complete independence and 4 points indicating complete dependence on others.
- (5) Data on walking parameters and joint and muscle function indicators of all subjects were collected, including walking frequency, walking speed, single leg weight-bearing time (SLWT), and knee and hip joint flexion and extension range of motion.
- (6) Data on serum inflammatory factor indicators of all subjects were collected.

### Quality control

To ensure the quality of the study and the accuracy of the data, the selection of all subjects and treatment plans was strictly carried out according to unified standards to ensure comparability between groups. During data collection, trained professionals performed all assessments using standardised operating procedures, and regular data audits were conducted to prevent omissions or errors. Throughout the treatment process, the rehabilitation plans for the EG and CG were implemented by experienced rehabilitation teams to ensure the standardisation and personalisation of treatment. In addition, the experiment received approval from the ethics committee and was conducted with the informed consent of the subjects to ensure that the experimental process met ethical requirements.

### Statistical processing

SPSS 22.0 statistical software was adopted. Measurement data conforming to normal distribution were presented by mean ± sd (x̄±s), and categorical data were presented by frequency and percentage (%). Non-normally distributed measurement data were analysed adopting the Mann-Whitney test, normally distributed measurement data were analysed adopting one-way ANOVA, and categorical data were analysed adopting the 2 test. P<0.05 were considered statistically meaningful.

## Results

### Contrast of paralysed parts and onset time of subjects

**Figure 1:**
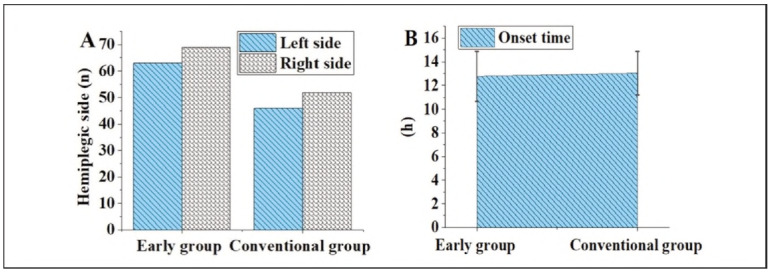
Contrast of the paralysed parts and onset time of subjects. (A for paralysed parts; B for onset time).

The paralysed parts and onset time of subjects are illustrated in Figure 1. The distinctions in paralysed parts and onset time between the EG and the CG were not statistically meaningful (P>0.05).

### Contrast of NIHSS scores before and following interference in subjects

**Figure 2:**
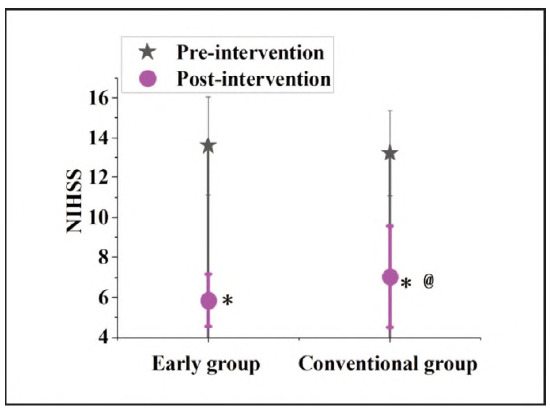
Contrast of NIHSS scores in subjects.

In Figure 2, before the intervention, the NIHSS score of the EG was 13.59±2.47, which decreased to 5.85±1.31 following interference. The CG was 13.22±2.16 and 7.03±2.54, respectively. No visible distinction was noted in the NIHSS scores of the subjects before intervention (P>0.05). After treatment, the NIHSS scores of the subjects were markedly lower; the NIHSS score of the EG was markedly lower than against the CG (P<0.05).

### Contrast of WGS scores before and following interference in subjects

**Figure 3:**
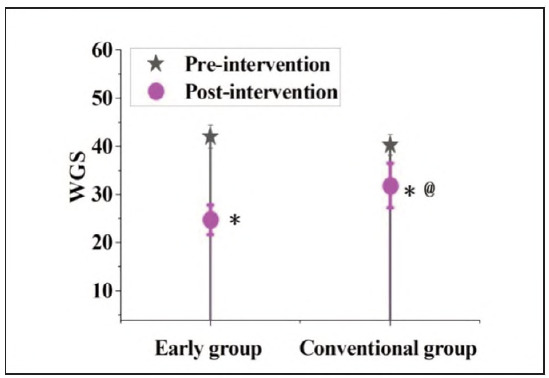
Contrast of WGS scores in subjects.

In Figure 3, before the intervention, the WGS score of the EG was 42.08±2.47, which decreased to 24.81±3.06 following interference. The CG was 40.33±2.16 and 31.96±4.62, respectively. No visible distinction was noted in the WGS scores of the subjects before intervention (P>0.05); however, following interference, the distinction in WGS scores was markedly different (P<0.05).

### Contrast of ADL scores before and following interference in subjects

**Figure 4:**
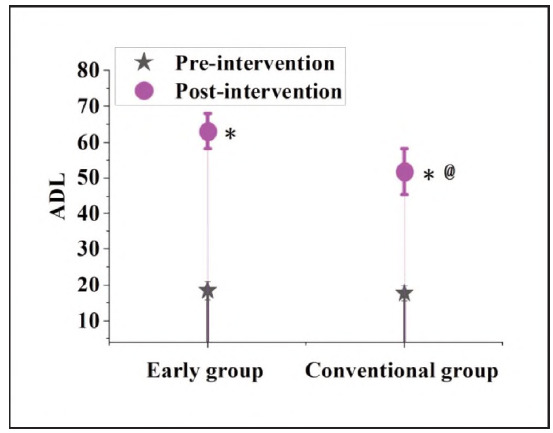
Contrast of ADL scores in subjects.

In Figure 4, before the intervention, the ADL score of the EG was 18.44±2.56, which increased to 63.08±4.93 following interference. The CG was 17.71±2.08 and 51.78±6.34, respectively. No visible distinction was noted in the ADL scores of the subjects before intervention (P>0.05). After treatment, the ADL scores of the subjects were markedly higher; the ADL score of the EG was markedly lower than against the CG (P<0.05).

### The contrast of walking frequency and walking speed before and following interference in subjects

**Figure 5:**
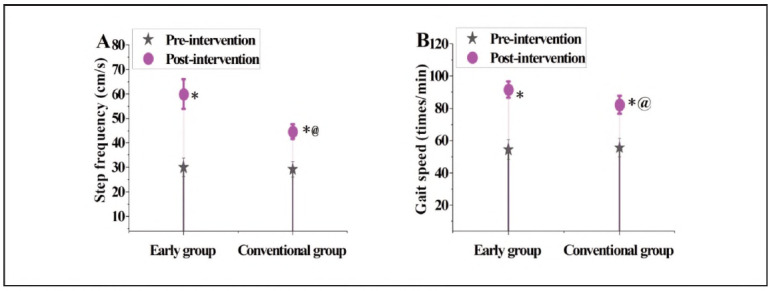
Contract of walking frequency and walking speed in subject.<br>Note: * means as against before intervention, @ means as against the EG, P<0.05

In Figure 5A and Figure 5B, in terms of walking frequency, the EG was 30.07±3.76 before intervention and markedly increased to 59.97±6.01 following interference; the CG was 29.31±3.14 and 44.58± 3.08, respectively. Regarding walking speed, the EG was 54.49±6.13 before intervention and improved to 91.56±4.92 following interference; the CG was 55.73±5.74 and 82.24±5.58, respectively. No visible distinction was noted in the subjects' walking frequency and walking speed before intervention (P>0.05). Still, following interference, the EG suggested markedly better walking frequency and walking speed than the CG (P<0.05).

### The contrast of joint and muscle function indicators before and following interference in subjects

**Figure 6:**
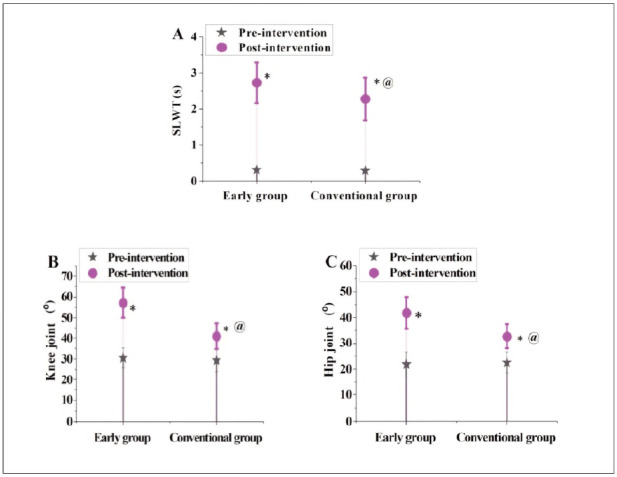
Contrast of SLWT, knee joint flexion and extension range of motion, and hip joint flexion and extension range of motion in subjects.<br>(A-C represent SLWT, knee joint flexion and extension range of motion, and hip joint flexion and extension range of motion, respectively) Note: * means as against before intervention, @ means as against the EG, P<0.05

In Figure 6A, Figure 6B, and Figure 6C, in terms of SLWT, the EG was 0.31±0.04s before intervention and markedly raised to 2.73 ±0.56s following interference; the CG was 0.29±0.06s and 2.28±0.59s, respectively. Regarding the range of motion of knee joint flexion and extension, the EG was 30.45±4.79° before intervention and obviously raised to 57.18±7.31° following interference; the CG was 29.31 ±5.33° and 40.99±6.22°, respectively. In terms of the range of motion of hip joint flexion and extension, the EG was 21.83±4.73° before intervention and improved to 41.78±6.11° following interference; the CG was 22.54±4.02° and 32.62±4.79°, respectively. No visible distinction was noted in SLWT, knee joint, and hip joint flexion and extension range of motion of the subjects before intervention (P>0.05). Following interference, the EG suggested obviously better SLWT, knee joint, and hip joint flexion and extension range of motion as against the CG (P<0.05).

### Contrast of serum inflammatory factors before and following interference in subjects

**Figure 7:**
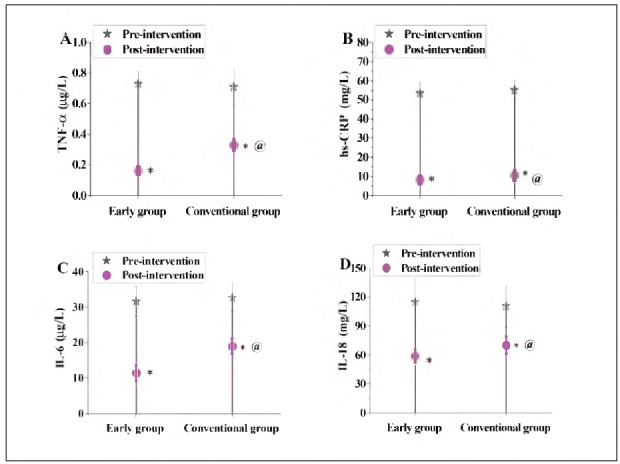
Contrast of serum inflammatory factors. (A-D: TNF-α, hs-CRf) IL-6, IL-18).<br>Note: * means compared with pre-intervention, @ means compared with EG, P<0.05

In Figure 7, no visible distinction was noted in the serum inflammatory factor levels of the subjects before intervention (P>0.05). Following interference, the serum inflammatory factor levels in the EG were obviously lower as against the CG (P<0.05).

## Discussion

In rehabilitation treatment for patients with AIS, enhancing therapeutic effects and accelerating neural repair have always been an important topic in clinical research [17]. The rehabilitation of hemiplegic patients after stroke is not only related to the recovery of limb function but is also closely associated with the patient's daily living abilities and QoL. Although traditional rehabilitation treatments have played a role in promoting recovery to some extent, they are usually based on standardised treatment plans and lack personalisation, quantification, and early intervention [18]. In recent years, with the development of neuroscience and the advancement of rehabilitation technology, new methods such as precision exercise rehabilitation, intelligent devices, and neurofunctional training have gradually gained clinical attention, especially in the application for patients in the acute phase [19]. This article compared the effects of early precision exercise rehabilitation with traditional rehabilitation to explore the impact of precision intervention on the rehabilitation of hemiplegic patients with AIS and to provide new ideas for the rehabilitation of stroke patients. The results of this article indicate that early precision exercise rehabilitation is obviously superior to traditional conventional rehabilitation in multiple assessment dimensions. Firstly, in terms of NIHSS scores, the scores following interference were obviously lower, and the post-treatment NIHSS scores of the EG were obviously lower than against the CG. This suggests that early precision exercise rehabilitation can more effectively improve neurological deficits and promote the recovery of patients' neurological functions [20]. The visible decrease in NIHSS scores, a commonly adopted tool to measure neurological deficits in patients with AIS, means the advantage of early precision intervention in accelerating neural repair. Secondly, in terms of gait assessment, the WGS scores of the EG following interference were obviously better than against the CG, indicating that early precision exercise rehabilitation can more effectively improve the stability, coordination, and fluidity of patients' gait.

In this article, the EG adopted technologies, including neurofunctional training, FES, and intelligent rehabilitation devices during the rehabilitation process. These treatment methods help promote neural plasticity in the cerebral cortex, accelerating neural repair. Regarding joint and muscle function indicators such as SLWT, knee joint flexion and extension range of motion, and hip joint flexion and extension range of motion, the EG suggested visible superiority over the CG in the above indicators following interference. These all indicate that early precision exercise rehabilitation treatment, through personalised intervention plans, can obviously improve patients' joint mobility and limb motor function [21] [22]. This article also assessed patients' serum inflammatory factors. The results suggested that no visible distinction was noted in the levels of inflammatory factors of the subjects before the intervention. Still, after treatment, the levels of serum inflammatory factors in the EG were lower than those of the CG. This result suggests that early precision exercise rehabilitation treatment can effectively suppress systemic inflammatory responses in the later stages of stroke [21]. The decrease in inflammatory factor levels may provide a more favourable microenvironment for neural repair, thereby accelerating the recovery of neurological functions. Existing studies have shown that neuroinflammatory responses after stroke are important factors affecting neural recovery and functional reconstruction. Excessive inflammatory responses exacerbate neural damage and may hinder neural self-repair. Therefore, early intervention is visible in promoting neural repair by regulating inflammatory responses.

## Conclusion

This study highlights the impact of early precision exercise rehabilitation on serum inflammatory cytokines and functional recovery in hemiplegic patients following acute ischemic stroke (AIS). The findings demonstrate that early intervention significantly improves neurological function, motor ability, gait performance, and self-care capacity compared to conventional rehabilitation. Additionally, it effectively reduces inflammatory cytokines (TNF-α, hs-CRP IL-6, IL-18), promoting neural repair and recovery. Integrating modern neuroscience-based rehabilitation strategies, including functional electrical stimulation, motor-evoked potential therapy, and intelligent rehabilitation devices, enhances treatment efficiency and precision. These results support the clinical adoption of early precision rehabilitation as a standard approach for optimising post-stroke recovery. Future research should explore long-term outcomes and individualised treatment protocols in diverse patient populations to refine rehabilitation strategies further.

## Dodatak

### Author contributions

All authors contributed to the study's conception and design. Hongxia Li performed material preparation, data collection, and analysis. Yunqi Lai wrote the first draft of the manuscript, and all authors commented on previous versions. All authors read and approved the final manuscript.

### Ethical approval statement

This retrospective chart review study involving human participants was conducted according to the ethical standards of the institutional and national research committee, along with the 1964 Helsinki Declaration and its later amendments or comparable ethical standards. The Human Investigation Committee (IRB) of Zhejiang Rehabilitation Hospital approved this study (Approval number: ZRH021).

### Informed consent

The authors affirm that human research participants provided informed consent to publish the images in Figure 1A, 1B and 1C. The participant consented to submitting the case report to the journal. Patients signed informed consent regarding publishing their data and photographs.

### Availability of data and materials

The datasets generated during and/or analysed during the current study are available from the corresponding author upon reasonable request.

### Conflict of interest statement

All the authors declare that they have no conflict of interest in this work.
